# Mechanism and Treatment Related to Oxidative Stress in Neonatal Hypoxic-Ischemic Encephalopathy

**DOI:** 10.3389/fnmol.2019.00088

**Published:** 2019-04-11

**Authors:** Xingping Qin, Jing Cheng, Yi Zhong, Omer Kamal Mahgoub, Farhana Akter, Yanqin Fan, Mohammed Aldughaim, Qiurong Xie, Lingxia Qin, Lijuan Gu, Zhihong Jian, Xiaoxing Xiong, Renzhong Liu

**Affiliations:** ^1^Department of Neurosurgery, Renmin Hospital of Wuhan University, Wuhan, China; ^2^Department of Neurosurgery, Harvard Medical School, Boston, MA, United States; ^3^Department of Neuroscience, University of Cambridge, Cambridge, United Kingdom; ^4^Central Laboratory, Renmin Hospital of Wuhan University, Wuhan, China; ^5^Department of Gynecology and Obstetrics, Renmin Hospital of Wuhan University, Wuhan, China; ^6^Department of Neurology, Renmin Hospital of Wuhan University, Wuhan, China

**Keywords:** neonatal hypoxic ischemic encephalopathy, mechanisms and therapy, reactive oxygen species, oxidative stress, clinical biomarkers, antioxidant therapy, mitochondria

## Abstract

Hypoxic ischemic encephalopathy (HIE) is a type of neonatal brain injury, which occurs due to lack of supply and oxygen deprivation to the brain. It is associated with a high morbidity and mortality rate. There are several therapeutic strategies that can be used to improve outcomes in patients with HIE. These include cell therapies such as marrow mesenchymal stem cells (MSCs) and umbilical cord blood stem cells (UCBCs), which are being incorporated into the new protocols for the prevention of ischemic brain damage. The focus of this review is to discuss the mechanism of oxidative stress in HIE and summarize the current available treatments for HIE. We hope that a better understanding of the relationship between oxidative stress and HIE will provide new insights on the potential therapy of this devastating condition.

## Introduction

Hypoxic ischemic encephalopathy (HIE) is a severe brain injury which occurs due to intrapartum asphyxia (Gluckman et al., 2005; Shankaran et al., 2005; Azzopardi et al., 2009). Neonatal HIE accounts for approximately 25% of global neonatal deaths (Liu et al., 2015b; Tagin et al., 2015). It has an annual incidence of 1.5 of every 1000 newborns (Kurinczuk et al., 2010), with a greater incidence in in developing countries (Azra Haider and Bhutta, 2006).

Neonates with HIE have greater incidence of cerebral palsy, cognitive impairment, growth restriction, and epilepsy (Cowan et al., 2003; Badawi et al., 2005; Lee et al., 2005). Magnetic resonance imaging (MRI) studies show that perinatal hypoxic-brain injury is characterized by structural damage to the gray matter (e.g., the basal ganglia, the thalamus, and the cortex) and to a less extent infarctions in white matter. Extensive structural damage is associated with worse motor outcomes and neurodevelopment delay (Rutherford et al., 2010a,b; Alderliesten et al., 2013).

Brain ischemia initiates a cascade of biochemical events as a result of a loss of adenosine triphosphate (ATP) and a failure of ATP-dependent ion transport pumps, leading to influx of sodium (Na^+^) and calcium (Ca^2+^) into cells. There is also a pump failure exporting Ca^2+^ out of the cell resulting in release of neurotransmitters such as glutamate, which stimulates α-amino-3-hydroxyl-5-methyl-4-isoxazole-propionate (AMPA) and *N*-methyl-D-aspartate (NMDA) receptors (Arteaga et al., 2015; Davidson et al., 2015; Wu et al., 2015), leading to excitotoxicity of cells and generation of free radicals. These can initiate elements of the programmed cell death cascade via redox signaling damaging DNA, proteins, and lipids, and also compromising the integrity of the blood–brain barrier.

Studies from small and large animal models have shown that as pregnancy progresses, developing brains become increasingly vulnerable to HIE, due to the brain’s high metabolic needs, leading to neuronal cell death (Volpe, 2001; Low, 2004; Vannucci and Hagberg, 2004; Bryce et al., 2005). Following HIE, cells initially experience impaired cerebral oxidative metabolism, swelling, and accumulation of extra-cellular excitatory amino acids, followed by a brief recovery before secondary energy failure ensues. Once reoxygenation takes place, reperfusion injury occurs leading to neuron cell death. Reperfusion injury includes irritant toxicity, deterioration of mitochondrial function, inflammation, activation of nitric oxide synthase (NOS), production of reactive oxygen species (ROS), and intracellular Ca^2+^ accumulation (Aridas et al., 2014; Arteaga et al., 2015; Davidson et al., 2015; Wu et al., 2015).

## Oxidative Stress and Neonatal Brain Injury

**FIGURE 1:**
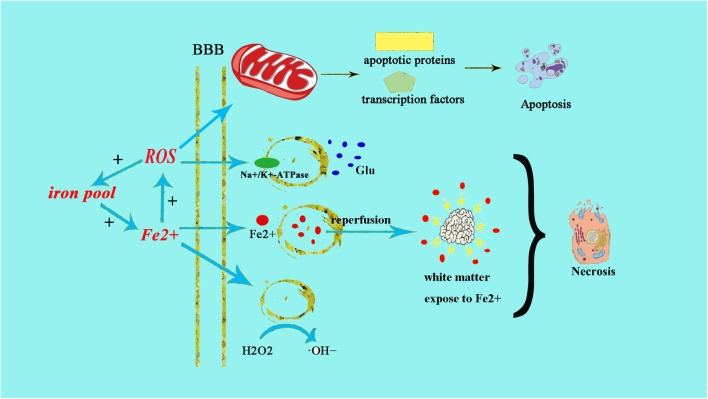
Fe^2+^ production after HIE result in neonatal brain cell injury. The excessive production of Fe^2+^ after HIE leads to increased production of ROS and OH–. The increased production of ROS can directly cause endothelial cell damage, the same as ⋅OH–. Fe^2+^ can also directly damage the Na^+^/K^+^-ATPase and lead to the depolarization of brain cells. Then brain cells release a large amount of glutamate and produce excitotoxicity, resulting in cell damage afterward. Meanwhile, the increased glutamate can enter into cell to exchange cystine, cystine deficiency activate cell apoptosis, and cause cell death by oxidative stress.

Neonatal brains are more susceptible to oxidative stress for a number of reasons: (1) neonatal brain is rich in fatty acids which make it particularly vulnerable to free radicals and lipid peroxidation insults; (2) compared to adults, the newborn’s brain is rich in iron (Fe^2+^), which plays a crucial role in growth and development. However, unbound iron can contribute to the development of free radicals. Free ions also catalyze the formation of ROS. When the newborn’s brain is exposed to Fe^2+^ ions, hydrogen peroxide (H_2_O_2_) can catalyze the formation of hydroxide (OH) ion, which is more oxidative, thus resulting in a more severe brain damage (Aridas et al., 2014) (Figure 1); (3) the cellular structure of the immature brain has its own characteristics in different stages. The periventricular white matter in preterm infants is characterized by preferential death of immature oligodendrocytes (Ferriero, 2001), which are particularly sensitive to oxidative damage mediated by free radicals. In term infants, the basal ganglia and thalamus, which are abundant in glutamate receptors allowing glutamate binding, are prone to damage during a hypoxic-ischemic insult (Aridas et al., 2014); (4) protogenic cells of immature brain oligodendrocytes are highly sensitive to free radicals while mature brain is more tolerant of oxidative stress. Some experiments have found that the half maximal effective concentration required to scavenge 50% of H_2_O_2_ (EC50 value) in the immature brain was lower than in the mature brain (Ferriero, 2001); (5) the antioxidant function of immature brain tissue is imperfect. Various studies (Baud et al., 2004) have indicated that the catalase activity in the antioxidant enzyme system is increased by twofold during embryonic day 18 and day 1 after birth, glutathione peroxidase (GPx) activity is increased by threefold, and GSH content is also significantly increased. Oxidative stress activates xanthine oxidase, which accumulates in the endothelium of the cerebral capillaries, therefore exposing the blood–brain barrier to oxidative stress (Khan and Black, 2003). This will result in free radical peroxidation of cell membranes to induce the production of inflammatory cytokines and promote leukocyte adhesion to the microvascular endothelium, allowing these leukocytes to cause further damage to endothelial cells (Buonocore et al., 2001). In this way, under the action of ROS, the integrity of the blood vessel is damaged, leading to intracranial hemorrhage following neonatal oxidative stress. Free radicals and their derivatives damage brain cells by producing excessive Fe^2+^ (Khan and Black, 2003), during hypoxia-ischemia, Fe^2+^ is released from the iron pool to produce free radicals, and the free radicals can release more Fe^2+^. Fe^2+^ in the plasma leak through the damaged blood–brain barrier into the brain and are then absorbed directly by brain cells. Fe^2+^ released into the extracellular space after ischemia-reperfusion result in damage to the periventricular white matter. Moreover, the free radicals impair transmembrane Na^+^/K^+^-ATPase activity, initiate depolarization of cell membrane and releasing a large amount of glutamic acid. Glutamate exchanges with cystine when entering the cell, leading to oxidative stress and cell death (Khan and Black, 2003). Free radicals then activate a series of transcription factors and apoptotic proteins through the mitochondrial pathway causing apoptosis of cell death (Figure 1).

## Mechanism of Oxidative Stress in HIE

**FIGURE 2:**
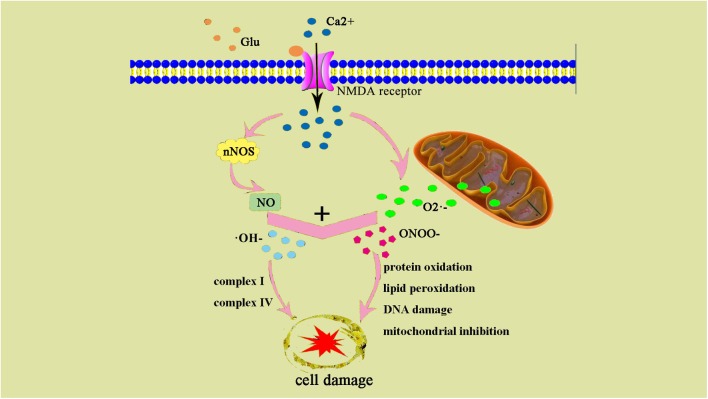
ONOO– and damage cell schematic after the occurrence of HIE generation. After the occurrence of HIE, a large amount of Ca^2+^ internal flow leads to NOS generates NO, NO, and superoxide O2– generate ONOO–, an excessive amount of ONOO– can also result in an increase of ⋅OH–. ONOO– and ⋅OH– lead to protein changes and degeneration, they also contribute to lipid peroxidation and DNA damage.

Oxidative stress leads to increased production of ROS and reactive nitrogen species (RNS). Low concentrations of ROS and RNS can be utilized as signaling molecules in normal physiological conditions (Wong and Crack, 2008). However, during hypoxic-ischemic injury, free radicals are produced rapidly. NMDA glutamate receptors stimulate synthesis of nitric oxide (NO) in the brain via activation of nitric oxide synthase (NOS). NO can react with superoxide, which is generated by the electron leak from the respiratory chain, particularly complexes I and III of the electron transport chain, to form peroxynitrite (ONOO-) and hydroxyl radicals (OH-). Subsequent OH-mediated cell injury occurs via protein oxidation, lipid peroxidation, DNA damage, and mitochondrial inhibition (especially complex IV). Complex I and IV are also targets of ONOO-direct damage (Figure 2).

The NADPH oxidase (NOX) family is associated with a large variety of ischemic and ROS-mediated vascular diseases. The NOXs and their catalytic subunits (NOX) are unique in their role as sole enzymes responsible for ROS generation. The relevant enzymatic isoforms which are associated with vasculopathies are NOX1, NOX2, and NOX4. Genetic mutations which are associated with a loss of NOX1 functionality have been shown to lower the occurrence of ROS-induced retinopathy in mouse models. Excitotoxicity-mediated calcium channels also cause the formation of O_2_ via the action of NOX (Weidinger and Kozlov, 2015). O_2_ can be neutralized by superoxide dismutase (SOD), which transform O_2_ into hydrogen peroxide (H_2_O_2_), and then is subsequently converted into H_2_O by catalase. However, the accumulation of excess H_2_O_2_ can lead to a further generation of OH- by iron-based Fenton reaction.

Oxidative stress is known to interact with the inflammatory system and create a “susceptibility window” for HIE. In the first step, it is activated by the residential immune cells such as microglia. Microglia strongly responds to hypoxic-ischemic attacks and is activated by increase in NO, glutamate, and ROS, producing a mass of inflammatory cytokines, such as IL-1β or TNF-α (Kaur et al., 2013; Knox et al., 2014). There is also activation of astrocytes, generating inflammatory cytokines, such as IL-1α, IL-1β, IL-6, TNF-α, and IFN-γ, which induces neuronal apoptosis, inhibits neurogenesis, and induces a direct damage of immune cells (Jellema et al., 2013). The mixture of cytokines, lymphocytes, and neutrophils exacerbate the damage of brain tissue. During the ischemia stage, neutrophils exacerbate brain damage though ROS production, decreased microvascular flow, and release of cytotoxic agents. Lymphocytes are thought to exacerbate brain injury, however, are thought to have limited role in inflammation in the neonatal brain (Tuttolomondo et al., 2008; Wang and Lu, 2008; Brait et al., 2010).

Immature brain has high oxygen consumption, contains high concentrations of Fe^2+^, is rich in water, and easily oxidizes unsaturated fatty acids. In addition, low-expression enzymes (SOD and GPx) of low myelination and antioxidants lead to an underdeveloped antioxidant system, making them particularly vulnerable to any oxidative damage, both ROS and RNS strongly influence excitotoxicity, which lead to cell death and mitochondrial damage (Ferriero, 2001; Perrone et al., 2010; Ikonomidou and Kaindl, 2011; Mirabelli-Badenier et al., 2011; Becker, 2012; Ten and Starkov, 2012).

Nitric oxide synthase and mitochondrial electron leakage are considered to be major contributors to ROS/RNS in immature brains (Mayurasakorn et al., 2016; Zhao et al., 2016). Expression of hydroxyl free radicals and Fe^2+^ increases ischemic areas in neonatal rats following HIE and this is partially mediated by nitric oxide (Palmer et al., 1999; Blomgren and Hagberg, 2006). Endothelial NOS and nNOS increased significantly only within hours after HIE (van den Tweel et al., 2005; Lu et al., 2015).

Mitochondria, as the primary site of oxidative metabolism, produce ATP as the “home of energy” (Ergenekon and Gucuyener, 1998; Ablikim et al., 2014). During the process of HIE, oxidative stress plays an important role in mitochondrial changes, which including mitochondrial dysfunction, ischemic starvation, reperfusion-induced hyperactivation, and delayed neuronal death. Mitochondria play a great role in the production of ROS, they are also susceptible to oxidative stress leading to cell death (Figure 1) (Acin-Perez et al., 2010; Sanderson et al., 2013). Cytochrome C is a crucial step in the induction of apoptosis when it released from the mitochondria. In order for this to happen, it must be separated from the cardiolipin (which is rich in polyunsaturated fatty acids) inside the mitochondria. Numerous studies have suggested that an increased ROS production may promote the release of cytochrome C from the mitochondrial inner membrane as a catalyst for cardiolipin oxidation (Petrosillo et al., 2001, 2003; Turrens, 2003). Due to the increase of ROS during HIE, the tightly bounded or loosely coupled CytC were disrupted released in the mitochondrial membrane; Fourth, the pro-apoptotic proteins which including Bid, Bad, Bax, Bak, Bok, and Bim will be increased in numbers outside the mitochondrial membrane, then have bound to Apaf-1 forming the complex of Apaf-1/caspase-9/Sitka, which lastly inactivated caspase-3, leading to neuronal apoptosis and death (Kagan et al., 2005; Nakka et al., 2008).

## Oxidative Stress and Clinical Biomarkers of HIE

Providing tools that quickly and accurately diagnose oxidative stress and response can undoubtedly help improve the diagnosis and prognosis of newborns. It is often difficult to perform frequent venipuncture in newborn and therefore non-invasive diagnostic tools, which are cost effective and easy to use are sought (Hardwick et al., 2012). Recent changes in oxidative status over time following specific therapeutic interventions can be monitored non-invasively with repeated urinalysis (Biomarkers Definitions Working and Group, 2001; Kuligowski et al., 2014). Although highly reliable methods have been proposed to support multiple analytical assays, the use of different biological fluids in neonatology as oxidative stress biomarkers is limited due to the requirement for complex equipment, highly specialized technicians, and cost (Saugstad et al., 2008; Vento et al., 2009; Perlman et al., 2010; Kuligowski et al., 2015). The quantification of oxidative stress is based upon measurements of biomarkers, biological fluids, and tissues that reflect the risk of oxidative stress on lipids, proteins, and DNA or increase damage to macromolecules. Biomarkers for oxidative stress include prostaglandins and non-protein bound iron (NPBI) biomarkers, both of which can be considered as indicators of the prognosis of the disease (Buonocore et al., 2003; Milne et al., 2011; Kapadia et al., 2013). Oxidative stress can be quantified by measuring the levels of prostaglandin F(2alpha)-like compounds, which are derived from the free radical mediated, non-ezymatic oxidation of arachidonic acid (Tonni et al., 2014). The intermediates formed during this reaction undergo 5-exo-trig cyclization and secondary addition of oxygen molecules, forming a prostaglandin-like compound, which are then reduced to F2-isoprostanes (F2-isoPs) (Jahn et al., 2008; Leung et al., 2015). These products are more stable and can thus be more easily detected in biological fluids (Galano et al., 2013). F2-IsoPs can be detected using gas chromatography and mass spectrometry. Other prostanes, which can also be used as reliable markers of oxidative damage, include F3-isoPs derived from peroxidation of eicosapentaenoic acid (EPA) (Kapadia et al., 2013). Currently, there is scant literature providing evidence of the relationship between oxidative stress indicators and the degree of brain damage and therefore needs to be explored further.

## Treatment of Neonatal Oxidative Stress Brain Injury

There are numerous therapeutic strategies for neonatal oxidative stress. These include mitochondrial therapy, free radical production inhibition, excitatory amino acid antagonists, nitric oxide inhibition, stem cell therapy, therapeutic hypothermia, and hyperbaric oxygen therapy.

### Mitochondrial Therapy

Much attention has been paid to the mitochondrial protein targets to keep a normal mitochondrial function. Although numerous therapies have been approved for clinical trials, current treatment strategies remain imperfect. There has been an increased interest in the use of small molecule inhibitors and activators that are targeted to mitochondrion (Leaw et al., 2017) tetra- and triphenylphosphonium (TPP), as well as multi-modal delivery of mitochondria-penetrating peptides (Smith et al., 2011; Silachev et al., 2015). Targeting bioactive therapeutics and antioxidants as an alternative to mitochondrion is gradually becoming a feasible method for the development of adjunct therapies.

Coenzyme Q10 (CoQ10) (a direct regulator of mitochondrial function) can reduce the product of ROS through the mitochondrial complex. CoQ10 have been found to be a neuroprotective agent in various animal models such as rodent models of Alzheimer’s disease (Li et al., 2007), middle cerebral artery occlusion (MCAO) (Belousova M. et al., 2016; Belousova M.A. et al., 2016) with or without hyperglycemia (Lu et al., 2017), and in traumatic brain injuries (Kalayci et al., 2011), However its’ role in neonatal hypoxic ischemic brain injury is yet to be explored.

Metformin have been proven to restrict mitochondrial respiration through a direct suppression of mitochondrial complex I in the respiratory chain (Owen et al., 2000; Andrzejewski et al., 2014). Metformin can act on inflammatory T cells by inhibiting the use of 2-deoxyglucose in glycolysis in rodent models of lupus, as well as inhibiting the production of interleukin 1β (IL1β) in LPS-induced sepsis (Carey et al., 2015). In the rat model of metabolic syndrome, maternal metformin treatment does well in preventing fetal inflammation, which is a risk factor for perinatal HIE (Desai et al., 2013). By applying an HIE rat model, metformin administration in neonatal brain is beneficial for the oligodendrocyte survival with consequent amelioration of HIE-induced myelination and behavior deficits (Owen et al., 2000).

Mitoquinone (MitoQ) is a potent and an effective antioxidant used to treat structural mitochondrial l damage, leading to an inhibition of lipid peroxidation and reduction of superoxide production as well as further ROS generation (Maguire et al., 1989; Ernster et al., 1992; Kelso et al., 2001; Reddy, 2006). MitoQ is a known ubiquinone derivative conjugated to TPP cation. In rat models of ischemia-reperfusion injury, MitoQ has shown a protective effect (Adlam et al., 2005). MitoQ had reduced the increased left ventricular pressure in perfused rat hearts that were subjected to ischemic injury and had a beneficial effects on respiratory control ratio, complex I, and aconitase activity (Adlam et al., 2005; Milagros Rocha and Victor, 2007). MitoQ may be a suitable treatment option in cerebrovascular diseases (CVDs) by reducing mitochondrial oxidative injury. The role of MitoQ in hypoxic-ischemic injury in the brain is not fully known (Ham and Raju, 2017); however, it may have a neuroprotective role in the thalamus (Hobbs et al., 2008).

### Free Radical Production Inhibitors and Free Radical Scavengers

Allopurinol is a specific inhibitor of xanthine oxidase, which can competitively inhibit xanthine oxidase and reduce the produce of oxygen free radicals in reperfusion. Allopurinol consumed by pregnant mothers leads to reduction in the level of S-100B protein (a marker of brain injury in cord blood) in the infant (Casetta et al., 2012). It has been shown that allopurinol serves as a chelator for NPBI and direct scavenger for free radicals, and thus may play a role in HIE treatment (Torrance et al., 2009; Kaandorp et al., 2010; Buonocore et al., 2012; Chaudhari and McGuire, 2012).

Deferoxamine is an iron chelator, which binds free iron and enhances its elimination. Deferoxamine crosses the blood–brain barrier and chelates non-bound protein iron (NBPI). In animal models of hypoxia-ischemia, it can reduce severity of brain injury and improve cerebral metabolism (Palmer, 1995).

Erythropoietin (EPO) is required for normal brain development. EPO increases the expression of anti-apoptotic genes, inhibits inflammation, reduces oxygen free radicals, decreases caspases protease activation, upregulates the PI3K/AKT, STAT5, and ERK signaling pathways (Peeters-Scholte et al., 2003; Park et al., 2006; Robertson et al., 2012).

Melatonin is a potent endogenous indoleamine, which has anti-oxidant, anti-inflammatory, and anti-apoptotic effects in HIE. Melatonin can freely cross blood–brain barrier and is thus attractive as a therapeutic agent for HIE (Lee et al., 2006; Carloni et al., 2008; Alonso-Alconada et al., 2013).

*N*-acetylserotonin (NAC)is a precursor of glutathione synthesis and an effective sulfhydryl-containing antioxidant. It is a scavenger of oxygen free radicals, increases glutathione levels, reduces redox potential, decreases cell death, reduces inflammatory cytokines, and inhibits NOS (Khan et al., 2004; Huang et al., 2008; Robertson et al., 2013). NAC attenuates hypoxi-ischemic brain injury in neonatal rats (Sekhon et al., 2003) and can be combined with systemic hypothermia to reduce brain injury (Park et al., 2015; Nie et al., 2016). NAC can attenuate demyelination in the corpus callosum, a white matter region which is particularly vulnerable to HIE (Jatana et al., 2006). Daily NAC (50 mg/kg) administration in rats in combination with hypothermia has been shown to increase myelin expression (Nie et al., 2016), improved brain function (Nie et al., 2016), and long-term neuromotor outcome (Park et al., 2015).

Superoxide dismutase is a radical scavenging enzyme, which may be useful in reducing brain injury; however, its applications are limited to poor permeability across the BBB. SOD encapsulated with biodegradable poly (D, L-lactide co-glycolide) nanoparticles (SOD-NPs) has been shown to effectively reduce brain infarct volume, edema, ROS formation, and neuroprotection in a rat model of cerebral ischemia-reperfusion injury (Reddy and Labhasetwar, 2009).

### The Role of Nitric Oxide

Nitric oxide may be either neuroprotective or neurotoxic during hypoxia-ischemia brain injury. Excessive NO production can lead to excitotoxicity, apoptosis, and inflammation. NO produced from endothelial NOS can play a neuroprotective role by preventing neuronal injury and maintaining cerebral blood flow. However, increased endothelial nitric oxide production can also promote endothelial dysfunction (Liu and Mu, 2014; Liu et al., 2015a,c).

### Magnesium Therapy

Magnesium is an attractive therapeutic agent for HIE due to its numerous neuroprotective effects. Magnesium is a NMDA receptor antagonist and prevents excitotoxicity by reducing calcium or glutamate. Magnesium also reduces inflammation and oxidative stress (McCord, 1985).

### Stem Cell Therapy

**FIGURE 3:**
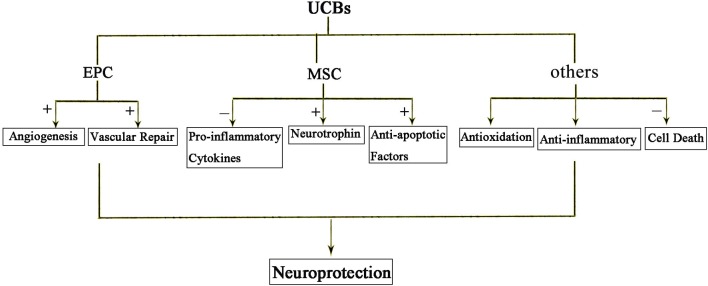
The protective effects of UCBs following HIE. Injection of cord blood stem cells has neuroprotective effects. Among them, EPC mainly promotes angiogenesis and repair. MSC acts through anti-inflammatory, secreted neurotrophic factors and anti-apoptotic factors. In short, anti-inflammatory and anti-oxidation and prevention of cell death are the three main mechanisms of action of cord blood stem cells.

Recent studies suggest bone marrow derived mesenchymal stem cells (BM-MSCs) and umbilical cord blood derived mesenchymal stem cells (UCB-MSCs) may play a role in HIE. Cell therapies, in particular, the cord blood stem is thought to provide protection from inflammation, apoptosis, oxidative stress, enhance regeneration, immune regulation, and anti-inflammatory effect (Figure 3). It is well known that components of the umbilical cord blood have the most effective damage-mediated inflammation for the treatment of the brain. Specific populations of cells, such as endothelial progenitor cells (EPCs) and MSCs, which been found in umbilical cord blood and tissue, have been proved to have potential utility in reducing the inflammatory processes that result from brain damage. MSCs have a strong immunomodulatory effect that protects against neuroinflammatory cascades that are triggered by HIE (Wasielewski et al., 2012; Li et al., 2016; Paton et al., 2017). Umbilical cord blood stem cell (UCBC) administration can also reduce the insult of white matter damage through a combination of anti-inflammatory or any other treatment options in HIE (Nakka et al., 2008). It is expected that further preclinical studies will optimize treatment options and require clinical trials to demonstrate the efficacy and safety for patients.

### Hyperbaric Oxygen Therapy

Hyperbaric oxygen therapy refers to a method of treating the body to inhale pure oxygen or high concentrations of oxygen at a level higher than one standard atmosphere. The study found that hyperbaric oxygen therapy may be through the induction of brain tissue SOD expression, thereby enhancing the organs’ antioxidant stress injury. However, the current application parameters of these therapeutic measures have not yet been unified, the timing of the application is still uncertain, and the exact effect still needs to be studied with multiple centers (Liu et al., 2013).

### Therapeutic Hypothermia

Therapeutic hypothermia can be classified according to the temperature as mild (32–35°C), moderate (28–32°C), and deep (<28°C). Induced moderate hypothermia is currently used as the mainstay therapeutic tool for HIE, validated by both clinical trials and experimental evidence. However, the long-term success of this treatment with regards to outcomes is inconclusive. Reduction in brain temperature enhances maintenance of high energy ATP stores during hypoxia-ischemia. It also normalizes protein synthesis and modulates activation of microglia and cytokines (Gulczynska and Gadzinowski, 2012). Therapeutic hypothermia leads to a decrease in cerebral metabolism by 5% for every degree Celsius reduction in temperature (Bain et al., 2014). Experimental studies in animals have shown that hypothermia can lead to reduction in neuronal cell death by reducing release of excitatory amino acids such as glutamate and reducing production of nitric oxide and free radicals (Wassink et al., 2014).

Several randomized controlled trials show that moderate therapeutic hypothermia is beneficial for attenuating neurodevelopment delay and mortality rates when initiated at less than 6 h for infants at 36 weeks’ or later gestation. Therapeutic hypothermia is usually continued for 72 h, followed by gradual rewarming over 12 h (Gluckman et al., 2005; Azzopardi et al., 2009). Hypothermia initiated after 6 h may have benefit but there remains uncertainty regarding its effectiveness (Laptook et al., 2017). Inclusion criteria for patients suitable for treatment include newborns aged >35 weeks with evidence of asphyxia such as pH < 7.0 of cord blood, Apgar score of less than 5 in the first 10 min of life or requirement for mechanical ventilation after the first 10 min of life, and evidence of encephalopathy (Silveira and Procianoy, 2015).

The two most common methods of inducing hypothermia include whole body cooling using a cooling blanket and selective head cooling with mild systemic hypothermia. Several groups have conducted comparison studies to determine which method is preferable for HIE treatment. There appears to be a higher incidence of hypoxic-ischemic lesions on MRI from patients who received selective head cooling vs whole body cooling (Sarkar et al., 2012). However, there appear to be no significant differences when comparing multi-organ dysfunction in infants receiving either method of treatment. Whole body cooling appears to be more favorable at present although selective head cooling may be just as useful in infants with less severe EEG changes (Sarkar et al., 2009). However, further large randomized controlled trials must be conducted to identify the best method for reducing both mortality and neurodevelopmental delay in HIE.

## Conclusion

In short, the pathogenesis of neonatal brain injury is extremely complex, there is a growing concern about oxidative stress in neonatal brain damage due to the neonatal brain own characteristics being more susceptible to oxidative stress injury. As a result, there is not an exact and effective treatment for neonatal brain injury. This article focuses on the mechanism of oxidative stress in neonatal brain injury. It is hoped that it would help to improve the public’s understanding of neonatal brain injury and help to diagnose neonatal brain injury indicators, prevention, and treatment measures.

## Author Contributions

All authors listed have made a substantial, direct and intellectual contribution to the work, and approved it for publication.

## Conflict of Interest Statement

The authors declare that the research was conducted in the absence of any commercial or financial relationships that could be construed as a potential conflict of interest.
